# Severe Chagas disease in Ecuador: a countrywide geodemographic epidemiological analysis from 2011 to 2021

**DOI:** 10.3389/fpubh.2023.1172955

**Published:** 2023-04-18

**Authors:** Jorge Vásconez-González, Juan S. Izquierdo-Condoy, Raul Fernandez-Naranjo, Esteban Gamez-Rivera, Andrea Tello-De-la-Torre, Galo S. Guerrero-Castillo, Carlos Ruiz-Sosa, Esteban Ortiz-Prado

**Affiliations:** ^1^One Health Research Group, Faculty of Health Science, Universidad de Las Americas, Quito, Ecuador; ^2^Postgraduate in Gastroenterology and Digestive Endoscopy, Faculty of Medicine, Pontificia Universidad Católica del Ecuador, Quito, Ecuador

**Keywords:** Chagas disease, parasites, neglected disease, high altitude, tropical, *Trypanosoma cruzi* (*T. cruzi*)

## Abstract

**Background:**

Chagas disease is a neglected and often forgotten tropical disease caused by the *Trypanosoma cruzi*. This parasite can be transmitted through the direct contact of human skin with feces and urine of the triatomine insect. According to the World Health Organization (WHO), an estimated 6–7 million people are infected worldwide, killing at least 14,000 every year. The disease has been reported in 20 of the 24 provinces of Ecuador, with El Oro, Guayas, and Loja being the most affected.

**Methodology:**

We analyzed the morbidity and mortality rates of severe Chagas disease in Ecuador on a nationwide, population-based level. Hospitalization cases and deaths were also examined based on altitude, including low (< 2,500 m) and high (> 2,500 m) altitudes, according to the International Society. Data was retrieved from the National Institute of Statistics and Census hospital admissions and in-hospital mortality databases from 2011 to 2021.

**Results:**

A total of 118 patients have been hospitalized in Ecuador since 2011 due to Chagas disease. The overall in-hospital mortality rate was 69.4% (*N* = 82). Men have a higher incidence rate (4.8/1,000,000) than women, although women have a significantly higher mortality rate than men (6.9/1,000,000).

**Conclusion:**

Chagas disease is a severe parasitic condition that primarily affects rural and poorer areas of Ecuador. Men are more likely to be infected due to differences in work and sociocultural activities. Using average elevation data, we conducted a geodemographic analysis to assess incidence rates by altitude. Our findings indicate that the disease is more common at low and moderate altitudes, but recent increases in cases at higher altitudes suggest that environmental changes, such as global warming, could be driving the proliferation of disease-carrying vectors in previously unaffected areas.

## 1. Introduction

Chagas disease (CD) is a neglected and often forgotten anthropozoonotic disease, first described by Chagas (1). This vector-borne disease is caused by a protozoan euglenoid parasite named *Trypanosoma cruzi*, which enters the human body after close contact with feces or urine from its vector, the triatomine insect also known as the “kissing bug” (2). Triatomes are arthropods from the Reduviidae family, comprising 128 recognized species classified as 17 genera in five tribes (3–5).

This disease, also called American trypanosomiasis, is endemic to Latin America and has been described in at least 21 countries. Although some reports suggest that CD has spread, rarely in the United States, Canada, Europe, and the Eastern Mediterranean region (4), the cases reported in these countries come mostly from emigrants from endemic countries and from people who traveled to countries where the disease is endemic and ended up catching it (6, 7). According to the World Health Organization (WHO), approximately 70 million people live in areas of high risk of infection; at least 6 to 7 million people are infected by this parasite each year, and roughly 30,000 new cases, including 9,000 newborns are diagnosed during prenatal and post-natal periods and around 14,000 deaths are attributed to this disease each year (2).

Transmission of CD occurs through direct contact with feces and urine from the triatomine insect (8). Once this type of arthropod bites a human, it defecates and urinates near the lesion (9). The parasites enter the body when the human instinctively scratches the bite and tears the skin’s integrity, allowing the insect’s feces to enter the inner tissues (10). Although other forms of transmission have been described, such as vertical transmission (congenital), through blood transfusion and organ donation, and even via contaminated food, the most common mechanism is vector-borne in endemic countries (11–15).

Ecuador is one of the South American nations with the worst CD burden, according to WHO (16). It has been estimated that 2.5% of the population in Ecuador has CD, which is higher than the 1.6% regional average (2, 17). In this sense, it is crucial to study CD in Ecuador not only because, according to the WHO, more than 370,000 people could be at risk of being infected by this parasite but also because of the impact on public health and its consequences on the cardiovascular and digestive systems are evident (2, 17).

Between 2013 and 2019, and based on the data of the Epidemiological Surveillance System (SIVE) of the Ministry of Public Health, 439 confirmed cases of Chagas disease were reported in Ecuador, with chronic cases (*n* = 331, 75.4%) being more common than acute cases (*n* = 108, 24.6%) (18). The disease has been reported in 20 of the 24 provinces, with El Oro (*n* = 104, 23.69%), Guayas (*n* = 64, 14.58%), and Loja (*n* = 60, 13.67%) being the most affected (17). Chagas disease has a high incidence in rural communities, with houses featuring thatched roofs, adobe or cracked walls, and no electricity, providing a suitable environment for the triatomine insect to reproduce (19). In a study of *Triatoma dimidiata* in the southern coastal region of Ecuador, 72% of the insects were infected with trypanosomes, and 95:77 were collected inside homes (20).

This study aimed to analyze the epidemiological characteristics of severe Chagas based on hospital admission data as a proxy for the incidence and mortality of CD in Ecuador.

## 2. Materials and methods

### 2.1. Study design

We conducted a cross-sectional countrywide study to determine the demographic and spatial distribution patterns of CD in Ecuador using hospital discharge and in-hospital mortality data as a proxy of incidence and mortality from 2011 until 2021.

### 2.2. Sample and setting

The study was conducted in Ecuador, the smallest country in the Andean region in South America. The country is divided into four geographical regions: the coast, mountains, Amazon, and Galapagos Islands. The political division contains 24 provinces and 221 cantons (cantons are political subdivisions of a province). Despite its small geographic size, Ecuador has an important climatic diversity characterized by a humid tropical climate in transition zones toward the coast and the Amazon, semi-humid to humid in the inter-Andean zone, hot and dry in the inter-Andean valleys and cold in the high mountains above 3,000 m altitude (21). According to the National Institute of Statistics and Census (INEC) in 2022, the population of Ecuador was 18,034,344 inhabitants (22).

The hospitalization cases of CD were defined according to the operational definitions outlined by the Ministry of Health of Ecuador, which can be found in Supplementary file 1 (23).

### 2.3. Data source and description

All hospitalization cases of CD B560 (Trypanosomiasis due to *Trypanosoma cruzi*), B561 (South American trypanosomiasis), B572 (American trypanosomiasis), B5721/I412 (American trypanosomiasis with heart injury/American trypanosomiasis with specified organ damage, Not classified elsewhere), and B575 (Brazilian trypanosomiasis) according to the 3-digit ICD-10 classification, were retrieved from National Institute of Statistics and Census (INEC).

Continuous and categorical data were acquired at a national level. In addition, when available, data was collated for the above-diagnosed cases during 11 years from the 24 provinces and the 221 cantons in the country. The data in our study includes information from both public healthcare providers (such as those with universal coverage and social security pension plans) and private healthcare providers (including both for-profit and non-profit entities).

### 2.4. Data analysis

We analyzed the following variables: age, sex, place of residence, and year of hospital admission. The incidence, mortality, and case-fatality rates were sex-and-age-standardized using projection data by canton and province according to the 2010 census. Incidence was calculated by dividing the number of hospitalization cases per year by the total population at risk each year for every age group. The incidence and mortality rates were computed by age, sex, geographic location, and corresponding population. All cases were classified across 17 age groups. The incidence and mortality of CD by the altitude of residence were also analyzed. The classification of low altitude <2,500 m and high altitude >2,500 m was used as a cut-off point for exposure to altitude. The analysis was also carried out using the classification offered by the International Society of Mountain Medicine low altitude (<1,500 m), moderate altitude (1,500 m – 2,500 m), high altitude (2,500–3,500 m).

## 3. Results

One hundred eighteen patients were hospitalized in Ecuador from 2011 to 2021 due to CD. The overall in-hospital mortality rate was 69.4% (*N* = 82). Men have a higher incidence rate (4.8/1,000,000) than women, although women have a significantly higher mortality rate than men (6.9/1,000,000).

### 3.1. Age and sex analysis

The average age of analyzed cases was 61.49 years (SD 23.84) for men and 60.58 years (SD 25.14) for women. From 2011 to 2021, in Ecuador, the incidence of CD has had an oscillating behavior without a clear predominance for one of the sexes, represented by incidence rates ranging from 3/ 1,000,000 in the years with fewer cases to 12/1,000,000 in 2017 with the highest number of cases.

### 3.2. Incidence rates

The overall sex-specific adjusted incidence rate was higher for men (4.8/1,000,000). In the analysis by age group, among women, the most affected were those over 75 years of age; on the other hand, among men, it is observed that CD predominantly occurs in younger groups (65 years and older) (Table 1).

**Table 1:** Incidence and mortality rates per 1,000,000 population by sex and age–specific groups in Ecuador, from 2011 to 2021.

Age (years)	Women			Men		
	Cases (n)	Incidence rate	Mortality rate	Cases (n)	Incidence rate	Mortality rate
<1	–	–	–	1	5.8	–
1–4	1	1.5	–	2	1.4	–
5–9	–	–	–	2	1.2	–
10–14	3	1.9	–	1	1.2	–
15–19	1	1.3	–	1	1.3	1.3
20–24	1	1.4	–	1	1.5	1.4
25–29	1	1.5	–	1	1.4	–
30–34	–	–	–	1	1.6	1.8
40–44	2	1.8	–	3	3.5	2.3
45–49	3	2.2	–	9	3.8	2.4
50–54	1	2.4	2.5	2	3.0	2.7
55–59	3	3.0	3.1	6	4.6	5.0
60–64	6	7.2	3.4	4	4.3	8.1
65–69	3	4.6	4.2	3	8.3	5.6
70–74	2	5.9	11	6	11.3	11.6
75–79	4	8.8	9.0	14	18.4	13.5
>80	12	11.7	12.0	18	20.0	18.4
Total	43	3.7	6.9	75	4.8	5.5

### 3.3. Mortality rate

The overall mortality rate shows that women are more affected by CD [6.9/1,000,000 (5.1–8.7)]. However, among young populations (0–49 years), males are the only ones who suffer death from the disease. On the other hand, both groups share an increase in mortality rates as the age of the patients increases, reaching the highest rates among those older than 80 years (Male Mortality Rate = 12/1,000,000 (10.7–13.4); Female Mortality Rate = 18.4/1,000,000 (17.1–19.6).

### 3.4. Geographic distribution

#### 3.4.1. Trends by province

**Figure 1:**
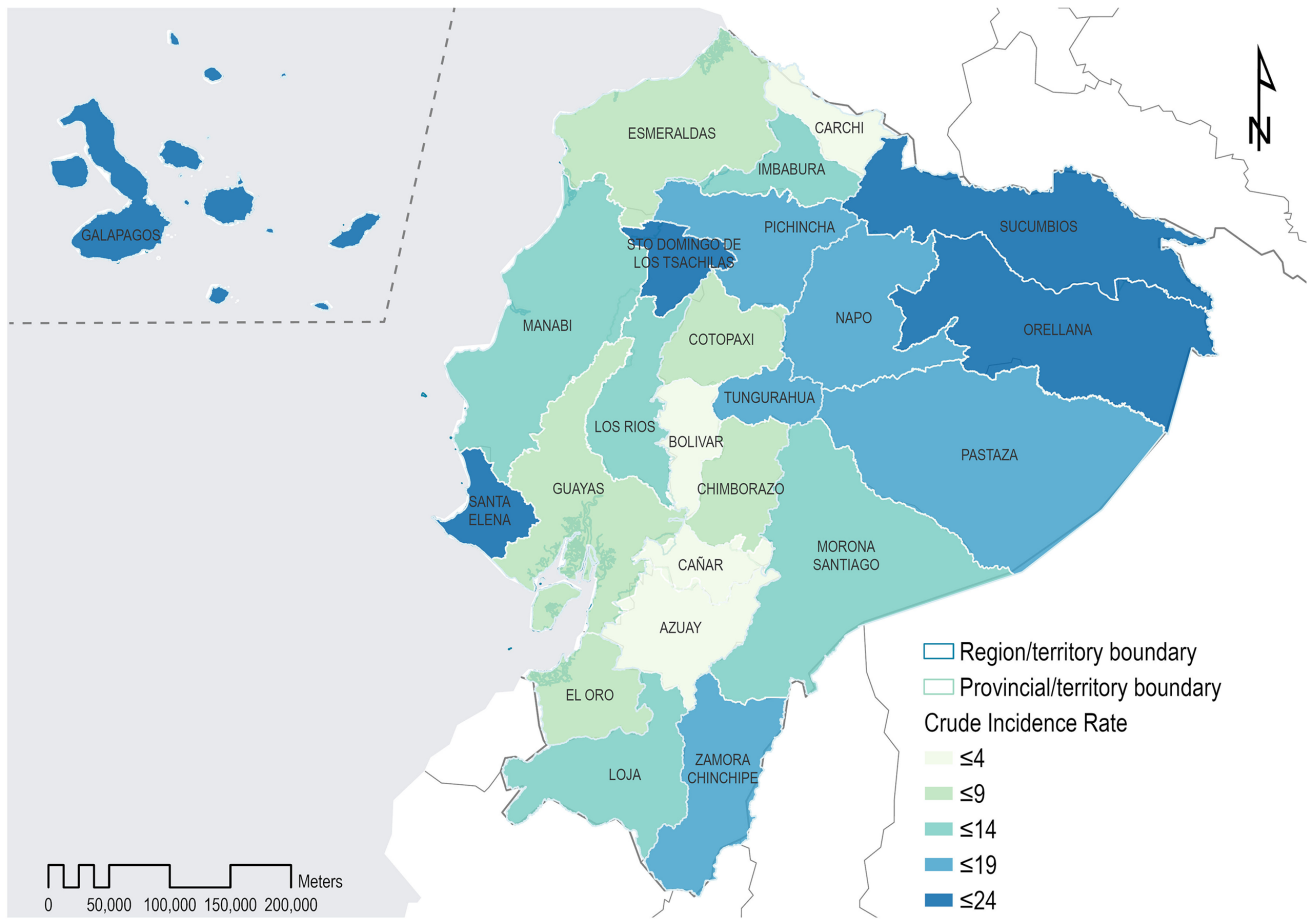
Geographical distribution of incidence rates of hospitalization cases for Chagas disease in the provinces of Ecuador, from 2011 to 2021.

Ecuador’s provinces with the highest Chagas adjusted incidence rates per 1,000,000 inhabits per the patient’s recorded residence are Zamora Chinchipe, El Oro, and Orellana, with 145.3, 116.3, and 78.2, respectively. On the contrary, Ecuador’s provinces with the lowest adjusted incidence rates are Pichincha, Guayas, and Manabí, with 5.3, 5.6, and 15.0, respectively (Figure 1 and Supplementary file 2).

According to mortality due to CD, Ecuador’s provinces with the highest adjusted mortality rates per 1,000,000 inhabits per the patient’s recorded residence are Orellana, El Oro, Santo Domingo De Los Tsachilas with 199.5, 156.2, and 58.8/1,000,000, respectively. On the contrary, Ecuador’s provinces with the lowest adjusted mortality rates are Pichincha, Guayas, and Los Rios, with 5.9, 8.1, and 17.3 per 1,000,000, respectively (Figure 1 and Supplementary file 2).

#### 3.4.2. Trends by canton

Ecuador’s cantons with the highest CD incidence rates per 1,000,000 population, as per the patient’s recorded residence, are Chinchipe, Atahualpa, and Chaguarpamba, with 687.1, 512.8, and 341.9, respectively. In contrast, Ecuador’s cantons with the lowest incidence rates are Quito, Guayaquil, and Cuenca, with 6.2, 9.6, and 7.3 per 1,000,000, respectively.

Ecuador’s cantons with the highest CD mortality rates per 1,000,000 population as per the patient’s recorded residence are Balsas, Portovelo, and Atahualpa, with 716.4, 532.72, and 509.16, respectively. On the contrary, Ecuador’s cantons with the lowest mortality rates are Quito, Guayaquil, and Cuenca, with 6.7, 12.5, and 49.9, respectively.

### 3.5. Altitude analysis

#### 3.5.1. Classic classification

Ecuador’s altitude regions with the highest CD adjusted incidence rates per 1,000,000 inhabits as per the patient’s recorded place of residence, were located at low altitudes (< 2,500 m) adjusted incidence rate (AIR): 33.88/1,000,000 compared to the highlanders (> 2,500 m) respectively (Table 2).

**Table 2:** Distribution of incidence, and mortality rates per 1,000,000 inhabits according to altitude ranges from 2011 to 2021.

Altitude range	Cases (n)	CIR	AIR	Deaths (n)	CMR	AMR
Low altitude <2,500 m	105	63.7	338.8	82	84.9	514.5
High altitude >2,500 m	13	16.4	137.0	5	8.1	19.9

CIR, crude incidence rate; AIR, adjusted incidence rate; CMR, crude mortality rate; AMR, adjusted mortality rate.

Related to deaths, the highest adjusted mortality rates per 1,000,000 inhabits were found in low altitudes (< 2,500 m) with adjusted mortality rate (AMR): 51.45/1,000,000 (Table 2).

#### 3.5.2. ISMM (International Society of Mountain Medicine) classification

Within this analysis, Ecuador’s altitude groups with the highest adjusted incidence rates per 1,000,000 inhabits were Moderate altitude (1,500–2,500 m) and low altitude (< 1,500 m), with 73.42 and 36.49, respectively. On the other hand, according to deaths, the adjusted mortality rates per 1,000,000 inhabits rates showed a trend like an incidence with AMR: 83.68 in Moderate altitude (1,500–2,500 m) and AMR: 57.51 in low landers (< 1,500 m) (Table 3).

**Table 3:** Distribution of incidence, and mortality rates per 1,000,000 inhabits according to ISMM altitude classification from 2011 to 2021.

Altitude range	Cases n	CIR	AIR	Deaths n	CMR	AMR
Low altitude (< 1,500 m)	98	61.5	364.9	76	83.4	575.1
Moderate altitude (1,500–2,500 m)	7	116.8	734.2	6	110.1	836.8
High altitude (2,500–3,500 m)	13	16.4	136.9	5	8.1	20.0

CIR, crude incidence rate; AIR, adjusted incidence rate; CMR, crude mortality rate; AMR, adjusted mortality rate.

## 4. Discussion

Chagas disease is a significant public health issue in Latin America, with an estimated 6–7 million people infected and approximately 65 million at risk of infection (2). The disease is endemic in 21 Latin American countries, where poverty, poor housing conditions, and inadequate healthcare infrastructure contribute to its high prevalence (24). In Ecuador, limited epidemiological research has been conducted despite active monitoring by the Minister of Public Health. While some studies have analyzed CD distribution in specific regions and provinces of the country, few comprehensive analyses are available. Our research underscores the ongoing burden of CD in Ecuador, particularly as revealed through hospitalization and mortality data recorded in the Ecuadorian Institute of Statistics and Census (INEC) public databases. Despite previous studies dating back over 60 years (25), there remains a dearth of research investigating the epidemiological impact of this vector-borne disease.

Our analysis revealed that Chagas disease hospitalizes an average of 15 patients annually in Ecuador. Notably, the incidence of hospitalization is highest among adults and older adults, likely due to the chronic and long-term effects of the disease on the heart and gastrointestinal system (26, 27). Regarding the severity of hospitalization cases, our findings suggest that most severe cases, as indicated by hospital mortality rates, occur in individuals over 70. In addition, our study revealed a higher mortality rate among adult men than women, a trend observed in other studies (28).

Additionally, our study highlights that CD cases are highest in provinces with warm climates and located at low or moderate altitudes, even after adjusting for the patient’s place of residence. On the other hand, provinces located in the highlands of Ecuador have lower rates of both incidence and mortality. These findings provide important insights for targeted interventions and resource allocation to address the burden of CD in Ecuador.

Research conducted in Ecuador, particularly in the field of entomology, has identified at least 17 species that have the potential to transmit *T. cruzi*. Among these species, nine are of particular concern, including *Triatoma dimidiata, Triatoma carrioni, Rhodnius pictipes, Rhodnius ecuadorensis, Rhodnius robustus, Panstrongylus rufotuberculatus, Panstrongylus chinai, Panstrongylus geniculatus*, and *Pastrongylus howardi* (29–31). These species are widely distributed throughout the country, taking advantage of Ecuador’s equatorial location and favorable climate conditions in the tropic of cancer. These findings underscore the importance of continued surveillance and control measures to prevent the spread of CD in Ecuador.

Castelle et al. reported that CD in Ecuador had been successfully controlled, as no new cases were reported in children under five in 2010 (32), However, subsequent studies indicate a different trend. Between 2013 and 2019, 10 cases were reported in children aged 1–4 years old and 11 cases in children aged 5–9. Additionally, two cases were reported in infants under 1 year old between 2015 and 2016 (33); Moreover, a study conducted in the Ecuadorian Amazon from 2015 to 2018, which analyzed the seroprevalence of CD, found nine cases in children aged 10 years old (34). In another study by Quinde-Calderón et al., which analyzed data from 2004 to 2014, a total of 915 human cases of CD were reported in Ecuador. Notably, there was a significant increase in reported cases over the years, followed by a decrease in 2013 and 2014 (35).

Our study observed that the number of hospitalizations for CD in Ecuador showed an increasing trend until 2017, followed by a decrease in subsequent years. However, our findings are inconsistent with the data from the Ministry of Public Health’s epidemiological surveillance system, which reported an increase in the number of CD cases from 2018 to 2021, with 74, 167, 113, and 170 cases reported each year, respectively (32). This suggests that there may be a gap between hospitalization data and disease surveillance data and highlights the need for further investigation into the epidemiology of CD in Ecuador.

Our study revealed higher incidence rates of CD among men in several age groups, consistent with the results of a similar study conducted in Mexico in 2018 (3) This trend can be explained by the higher involvement of men in agricultural activities during their productive years. However, our analysis also revealed that the overall mortality rate per 1,000,000 inhabitants was higher among women, despite higher mortality rates among men in most age groups. Previous studies in neighboring countries have yielded conflicting results on this variable. For instance, da Nóbrega et al. (36), found that 86% of deaths from CD in Brazil occurred in men and were associated with heart disease, while a 40-year investigation in Colombia showed that the highest mortality rates from CD were found in men over 65 years old (37). On the other hand, a systematic review with a meta-analysis conducted in 2016 did not find a significant difference in CD mortality related to sex (38).

We conducted a novel analysis to investigate the incidence and mortality of CD at different altitudes. Ecuador’s unique topography, with 221 cities situated at varying elevations ranging from sea level to 4,300 m, provides an exceptional opportunity to study the burden of diseases at different altitudes (39–41). Our study found that the majority of CD cases (*n* = 107) were concentrated at elevations below 2,500 m. To provide a more nuanced analysis, we also employed the International Society of Mountain Medicine classification, which classifies altitudes into different categories, and observed that the highest incidence and mortality rates were found at low and moderate altitudes. We hypothesize that this pattern may be attributed to the warmer temperatures that promote the growth and survival of Triatomes, as documented in prior research (42, 43). While our investigation did not explicitly aim to explore the relationship between climate change and the emergence of CD, our data suggest that the likelihood of discovering Triatomes at higher elevations may increase as temperatures continue to rise.

Our study highlights that ongoing efforts are crucial to control the spread of CD in Ecuador, particularly in provinces with warmer climates and low to moderate altitudes, where the disease incidence is concentrated. Strengthening disease control measures, such as implementing vector control strategies and health education programs, could reduce the burden of CD in these areas. Moreover, increasing funding and resources for research and surveillance programs could enhance our understanding of the disease and facilitate the development of more effective prevention and control measures (44–46).

This study establishes crucial precedents regarding the cost of caring for patients suffering from serious cardiovascular and gastrointestinal problems related to CD, such as cardiomegaly or megacolon, by using hospitalization data. Although several published studies in Ecuador exist, analyzing the epidemiological burden of different clinical presentations of CD is significant because it enables us to estimate the healthcare expenses incurred by the health system for treating these patients. On average, the Ecuadorian public healthcare system incurs a cost of over $300 per day of hospitalization, with patients staying in the hospital for three to 6 days, resulting in substantial financial losses for the Ministry of Public Health of Ecuador (47).

## 5. Limitations

When interpreting the results, one must consider several limitations of our study. These limitations include the observational and ecological study design, which limits the ability to establish causal relationships for the entire population. Additionally, the lack of information on the type of complication experienced by each hospitalized patient and the treatment given to them restricts the ability to draw specific conclusions about the disease burden of CD. Moreover, the nature of the data analyzed prevents the distinction between re-hospitalizations and single occasion hospitalizations.

Another limitation of our study is that we only included hospital discharge data and did not account for cases of CD that were mild or moderate and did not require hospitalization. This exclusion may have led to an underestimation of the actual disease burden and could have limited the generalizability of our findings to the entire population.

Despite these limitations, we believe that our study offers valuable insights into the burden of CD in Ecuador, particularly in terms of the incidence and mortality rates among different age and gender groups and at different altitudes. However, we need further research to obtain a more comprehensive understanding of the disease burden and to develop effective prevention and treatment strategies.

## Data availability statement

The datasets presented in this study can be found in online repositories. The names of the repository/repositories and accession number(s) can be found at: https://www.ecuadorencifras.gob.ec/camas-y-egresos-hospitalarios/.

## Author contributions

JV-G, JI-C, and EO-P contributed to the conception and design of the entire project, gained full access to data from the National Statistical Institutes in Ecuador, was primarily responsible for all aspects of the work, and ensure the completeness and accuracy of the investigation. RF-N, JI-C, EG-R, AT-D-l-T, GG-C, CR-S, and EO-P contributed to data acquisition and review of the available literature and initial writing of the manuscript. RF-N, JI-C, and EO-P contributed to the statistical analysis and internal validity of the study. JV-G, EG-R, AT-D-l-T, GG-C, and CR-S developed the draft version of the manuscript. JI-C and EO-P critically reviewed and edited the manuscript to its final complete version and provided input to the data report and its interpretation. All authors reviewed and approved the final version of the manuscript.

## Conflict of interest

The authors declare that the research was conducted in the absence of any commercial or financial relationships that could be construed as a potential conflict of interest.

## Publisher’s note

All claims expressed in this article are solely those of the authors and do not necessarily represent those of their affiliated organizations, or those of the publisher, the editors and the reviewers. Any product that may be evaluated in this article, or claim that may be made by its manufacturer, is not guaranteed or endorsed by the publisher.
