# Supplementary material for: Severe Chagas disease in Ecuador: a countrywide geodemographic epidemiological analysis from 2011 to 2021
Source: Front Public Health. 2023 Apr 18;11:1172955. doi: 10.3389/fpubh.2023.1172955 (PMC10151800; doi:10.3389/fpubh.2023.1172955)
Supplement: Supplementary file 1 [file Table_1.docx]

***Supplementary file 1.*** *Operational definitions of included Chagas disease cases.*

| **Category** | **Operational definition** |
| --- | --- |
| *Suspected acute Chagas case* | A person who lives in or has been in an endemic area in the last 2 months and presents with fever and one or more of the following signs or symptoms: Inoculation chagoma, Romaña's sign, lymphadenopathy, hepatosplenomegaly, rash, malaise, meningoencephalitis, cardiomyopathy, myocarditis. |
| *Confirmed acute Chagas* | Any suspected case of acute Chagas confirmed by the laboratory (thick smear test that detects the presence of the parasite in the blood or polymerase chain reaction (PCR). |
| *Suspected case of chronic Chagas* | The cases may or may not present the following symptoms: Patient with symptoms of dyspnea, heart failure, chronic heart lesions, or enlarged viscera or peripheral lung disease. Any case reactive to a screening test with or without signs or symptoms compatible with CD in the chronic stage is also considered a suspected case of chronic Chagas. |
| *Confirmed chronic Chagas* | Any suspected case of chronic Chagas confirmed by the laboratory (ELISA test: positive serology for IgG antibodies against T Cruzi by immunofluorescence or indirect hemagglutination or PCR). The average time for laboratory confirmation of cases is approximately 2 weeks. |

**Supplementary file 2**. Incidence and mortality rates per 1,000,000 inhabit caused by Chagas disease according to Ecuadorian provinces from 2011 to 2021.

| **Province** | **Cases (n)** | **CIR** | **AIR** | **Deaths (n)** | **CMR** | **AMR** |
| --- | --- | --- | --- | --- | --- | --- |
| Azuay | 5 | 31.2 | 31.2 | 2 | 36.2 | 36.0 |
| El Oro | 59 | 112 .1 | 116.2 | 47 | 145.5 | 156.2 |
| Guayas | 16 | 5.7 | 5.6 | 9 | 8.1 | 8.0 |
| Loja | 3 | 56.2 | 5.6 | 4 | 48.0 | 47.5 |
| Los Rios | 1 | 44.2 | 44.2 | 1 | 17.3 | 17.3 |
| Manabí | 13 | 15.4 | 15.0 | 10 | 19.6 | 2.0 |
| Morona Santiago | 2 | 77.7 | 76.4 | - | - | - |
| Orellana | 2 | 85.9 | 78.2 | 1 | 199.5 | 199.5 |
| Pichincha | 5 | 5.4 | 53.0 | 4 | 5.9 | 5.7 |
| Santa Elena | 3 | 50.3 | 50.3 | - | - | - |
| Santo Domingo T | 2 | 70.3 | 75.3 | 2 | 59.1 | 58.8 |
| Sucumbios | 3 | 63.8 | 64.4 | - | - | - |
| Tungurahua | 2 | 49.7 | 49.7 | - | - | - |
| Zamora Chinchipe | 2 | 160.3 | 145.3 | - | - | - |

CIR: crude incidence rate; AIR: adjusted incidence rate; CMR: crude mortality rate; AMR: adjusted mortality rate.
